# Pterostilbene Promotes Spinal Cord Injury Recovery by Inhibiting Ferroptosis via Keap1/Nrf2/SLC7A11/GPX4 Axis Activation

**DOI:** 10.3390/antiox15020188

**Published:** 2026-02-02

**Authors:** Yadan Dong, Yichen Liu, Yixuan Ji, Wen Meng, Xiaoxin Cheng, Xu Zheng

**Affiliations:** College of Basic Medical Science, Dalian Medical University, Dalian 116044, China; 15247442379@163.com (Y.D.); liuyichen1686@163.com (Y.L.); 17612280499@163.com (W.M.)

**Keywords:** pterostilbene, spinal cord injury, oligodendrocytes, ferroptosis, Keap1, Nrf2

## Abstract

**Background:** Spinal cord injury (SCI) represents a form of traumatic damage to the central nervous system, and oligodendrocytes play a central role in SCI recovery. Ferroptosis is a major factor in the pathophysiological development of SCI symptoms. Pterostilbene (Pte) has antioxidant, anti-inflammatory, and neuroprotective effects. This study aims to investigate the potential role of Pte in SCI. **Methods:** A SCI model of rats was constructed. The BBB score assessment, the footprint test, EC staining, immunofluorescence (IF), and Western blot (WB) were conducted to observe the neuroprotective effects of Pte. The factors of ferroptosis, such as Glutathione (GSH), Malondialdehyde (MDA), Fe^2+^, solute carrier family 7 member 11 (SLC7A11) and glutathione peroxidase 4 (GPX4), were assessed. Then, transcriptomic data, network pharmacology, molecular docking analysis, and the erastin-induced ferroptosis model of OLN-93 cell lines were used to investigate the mechanism of inhibiting ferroptosis by Pte. **Results:** Pte treatment restored motor function and spinal cord tissue in SCI rats. Furthermore, Pte dramatically decreased oligodendrocyte ferroptosis. Finally, we discovered that Pte can repair SCI by blocking ferroptosis via the Keap1/Nrf2/SLC7A11/GPX4 axis. **Conclusions:** Pte reduces lipid peroxidation via the Keap1/Nrf2/SLC7A11/GPX4 axis, which reduces the development of ferroptosis in oligodendrocytes and improves locomotor function in rats with SCI.

## 1. Introduction

Spinal cord injury (SCI) is a devastating traumatic disease of the central nervous system (CNS), characterized by the loss of motor and sensory functions, as well as autonomic nervous system dysfunction [1]. It not only seriously damages the physical and mental health of patients but also introduces a serious economic burden and social problems to families and society [2]. Although SCI causes serious consequences to the body, there are still limited effective treatment strategies for SCI due to its complex pathophysiological mechanisms [3,4]. The pathophysiology of SCI can be categorized into primary and secondary injuries. Primary injury results from direct mechanical trauma, such as vascular damage, axonal disruption, neuronal membrane rupture, and microhemorrhages. Secondary injuries result from the primary injury and include inflammatory responses, mitochondrial dysfunction, lipid peroxidation, free radical formation, and the activation of apoptosis signaling pathways [5]. One of the pathological changes associated with primary and secondary SCI is demyelination, which refers to the loss of myelin around axons at the lesion site due to the death of oligodendrocytes [6,7]. In the CNS, oligodendrocytes are specialized cells responsible for the axon formation of myelinated neurons [8]. The demyelination of neuronal axons caused by oligodendrocyte death after SCI is a key factor that aggravates axonal injury and regeneration difficulties [9,10,11]. Therefore, reducing oligodendrocyte death, thereby preventing axonal demyelination and promoting myelination of residual nerve fibers, is one of the key approaches to treating SCI.

Ferroptosis represents an iron-dependent type of regulated cell death, and it is distinguished by the lethal buildup of lipid peroxides [12,13]. To date, ferroptosis has been found to contribute significantly to the pathological progression of SCI. In the initial stage of SCI, hemorrhage occurs at the injured site, where a large number of erythrocytes and hemoglobin undergo breakdown and degradation, leading to a sharp increase in the iron level at the injured site. The excess iron in the injured spinal cord leads to the iron-mediated Fenton reaction, which produces substantial amounts of reactive oxygen species (ROS). In addition, the higher content of polyunsaturated fatty acids in the spinal cord makes it easier to generate lipid peroxides, which further promotes the ferroptosis of spinal nerve cells [14,15,16]. Research indicates that the ferroptosis inhibitor deferoxamine (DFO) and ferrostatin-1 (Fer-1) promote the repair and functional recovery of rats with spinal cord injury [17,18]. Additionally, the drugs targeting ferroptosis can also promote recovery from spinal cord injury [19,20,21,22]. These findings suggest that targeting ferroptosis may represent a novel therapeutic breakthrough for SCI.

In preventing ferroptosis, glutathione peroxidase 4 (GPX4) plays a crucial role. It uses glutathione (GSH) as a substrate, converting activated phospholipid hydroperoxide (PUFA-PLOOH) into inactivated PLOH. This process disrupts free radical chain reactions, inhibits lipid peroxidation, and suppresses ferroptosis. Additionally, the glutamate/cysteine antiporter system Xc- (System Xc-), composed of SLC7A11 and SLC3A2, mediates the intracellular transport of cysteine, thereby supplying raw materials for GSH synthesis [23,24]. Nuclear factor erythroid 2-related factor 2 (Nrf2), as a core regulatory element in the oxidative stress defense mechanism, plays a crucial role in preventing ferroptosis. Typically, Nrf2 is tightly regulated via the ubiquitinylation pathway mediated by the tumor suppressor protein Kelch-like ECH-associated protein 1 (Keap1), maintaining it at low levels. However, under oxidative stress conditions, Nrf2 is released from Keap1’s constraints, a process signifying its activation. Subsequently, Nrf2 translocates to the nucleus, triggering the transcriptional activity of a series of genes containing antioxidant response elements (AREs). These genes, such as SLC7A11 and GPX4, play crucial roles in inhibiting lipid peroxidation and preventing ferroptosis [25,26,27]. Therefore, the Keap1/Nrf2/SLC7A11/GPX4 pathway plays an important role in preventing ferroptosis.

Pterostilbene (Pte) is a natural dimethyl analogue derived from plants such as sandalwood, blueberries, and grapes, exhibiting pleiotropic therapeutic effects including anti-inflammatory, antioxidant, and neuroprotective activities [28,29,30]. Some studies have reported the inhibitory effect of Pte on oxidative stress and inflammation. For example, Pte inhibits the IL-β-induced inflammation and oxidative stress in chondrocytes via promoting Nrf2 nuclear translocation [31]. Pte activates the Nrf2-mediated antioxidant pathway in HaCaT cells, thereby reducing ROS levels after ultraviolet A (UVA) irradiation [32]. Additionally, Pte also exhibits antioxidant effects in the models of Alzheimer’s disease and amyotrophic lateral sclerosis (ALS) [33,34]. Notably, previous studies indicated that Pte could significantly alleviate ferroptosis in cardiomyocytes [35]. Currently, the effects and underlying mechanisms of Pte on oligodendrocyte ferroptosis after SCI remain largely elusive. In this study, we demonstrated that Pte treatment significantly improved histological and functional outcomes in SCI rats. Through in vitro and in vivo experiments, we further demonstrated that Pte significantly inhibited iron deposition and reduced lipid peroxides accumulation in oligodendrocytes. Our results indicate that Pte might be a promising therapeutic agent for the treatment of SCI.

## 2. Materials and Methods

### 2.1. Animal

Female SD rats (8 weeks old, 200–220 g) were obtained from Liaoning Changsheng Biotechnology Company (Benxi, China). Rats were employed in the present study to circumvent the bladder-related complications that are frequently seen in male rats following SCI. The rats were maintained under specific pathogen-free (SPF) conditions at 22 ± 2 °C, 55 ± 5% relative humidity, with a 12/12 h light/dark cycle and ad libitum access to water and food. All animal care and procedures were approved by Dalian Medical University, Institutional Animal Care and Use Committee (IACUC), with approval Number: AEE24052, 13 November 2024.

### 2.2. SCI Model and Pte Treatment

Adult female rats were anesthetized with sodium pentobarbital (30 mg/kg, intraperitoneal injection) (Sigma, St. Louis, MO, USA). The dorsal laminectomy was carried out at the ninth thoracic vertebra (T9) for spinal cord exposure, and then a moderate contusion SCI was performed with a Spinal Cord Contusion System Impactor M-III (diameter: 2.5 mm, weight: 10 g, height: 40 mm) (W.M.Keck Center for Collaborative Neuroscience, Rutgers, The State University of New Jersey, USA). Afterwards, the wound was sewn up in layers, and iodophor was applied to the wound site. Then, 0.2 mL of penicillin and streptomycin (Hyclone, South Logan, UT, USA) was injected intramuscularly. The bladders were manually emptied twice daily until the ability to urinate spontaneously was regained. Rats were assigned randomly into three groups: (1) a Sham group (*n* = 15), in which animals were subjected to laminectomy but did not receive SCI, but intraperitoneal injection contained, in equal parts, a 5% dimethyl sulfoxide (DMSO) (Sigma, St. Louis, MO, USA) in sterile saline solution for 1 week; (2) the SCI + Vehicle group (*n* = 15), in which animals received SCI without Pte treatment, and similarly, a sterile 5% DMSO solution with the same volume as Pte was accepted for 1 week; and (3) the SCI + Pte group (*n* = 15), in which animals received SCI and were treated with Pte (40 mg/kg, ip) daily for 1 week. Pte (HPLC ≥ 99%) (Dalian Meilun Biotechnology Co., Ltd., Dalian, China) was first prepared as a 500 mM stock solution using high-quality DMSO free of pyrogens. Aliquots were stored at −20 °C. Before use, the stock solution was dissolved in a sterile 0.9% saline solution to achieve a final DMSO concentration of 5%.

### 2.3. Behavioral Assessment

The functional performance of the hindlimb was investigated using the Basso–Beattie–Bresnahan (BBB) locomotor rating scale at 1, 3, 7, 14, 21, and 28 days after surgery. Rats were individually put in an open area featuring a non-sliding surface, in which each animal was allowed 5 min of free movement at a time. All rats were labeled with codes, and behavioral evaluations were conducted by two researchers who were blinded to the treatment groups.

Additionally, footprint analysis was tested at 7 and 28 days after surgery. The hind paws of the rats were marked with red paint. The rats were positioned on a sheet of paper and guided to walk in a straight line. The data of hindlimb coordination analysis were obtained by measuring the distance between adjacent fore-and-aft footprints on the same side.

### 2.4. Histological Analyses

The rats were briefly anesthetized using sodium pentobarbital, followed by transcardial perfusion with 0.01 M PBS (pH 7.4) and then perfused with 4% paraformaldehyde. Spinal cord segments containing the injured region were excised, immersed in a 30% sucrose buffer at 4 °C overnight, and then embedded in optimal cutting temperature (OCT) compound (Sakura, Torrance, CA, USA). Serial transverse sections (20 μm thickness) covering the whole injured region were prepared using a cryostat. Different batches of slides were stained using iron-eriochrome cyanine R (EC) and cresyl violet to distinguish myelinated white matter (WM) from residual spared tissue. Images of spinal cord sections at the epicenter and areas at 0.8 and 1.6 mm rostral and caudal from the epicenter, respectively, were observed using a microscope. The lesion epicenter was identified as the section with the minimal quantity of preserved myelinated WM. The proportion of preserved WM area was figured out by dividing the area of preserved WM by the total cross-sectional area of the spinal cord in the corresponding transverse section.

### 2.5. Perls Staining

A Perls Prussian blue staining kit (enhanced with DAB) (G1428, Solarbio, Beijing, China) was used to detect the amount of iron ions in tissues on the 7th day after spinal cord injury in the rats. Briefly, the spinal cord tissue was cut into 4 μm sections after paraffin embedding, and the sections were routinely deparaffinized and rehydrated. The sections were incubated in Perls staining solution for 20 min and then placed in incubation solution for 10 min. Signals were developed by incubation for 2 min in 3,3-diaminobenzidine (DAB), and the hematoxylin solution was used for counterstaining. Finally, the iron ion deposition at the epicenter in the perilesional area of spinal cord tissue was observed and photographed using a microscope.

### 2.6. Measurement of Malondialdehyde (MDA) Levels and Glutathione (GSH) Activity

An MDA assay kit (BC0025, Solarbio, Beijing, China) and a GSH activity assay kit (A006-2-1, Nanjing Jiancheng, Nanjing, China) were used to measure the levels of MDA and GSH in OLN-93 cells and spinal cord tissue extending 0.5 cm from the epicenter at both the rostral and caudal (total length 1 cm). MDA and GSH levels were calculated after protein concentrations were determined with the BCA Protein Assay Kit (P0012, Beyotime, Shanghai, China).

### 2.7. Screening Differentially Expressed Genes in SCI

We queried the Gene Expression Omnibus (GEO) database (https://www.ncbi.nlm.nih.gov/geo/) using the keyword “spinal cord injury” to screen for relevant gene expression datasets. We selected four transcriptome datasets containing the normal group and the 7-days-after-SCI group. They are GSE14317 (3 samples with non-SCI and 4 samples with SCI), GSE29488 (3 samples with non-SCI and 3 samples with SCI), GSE45006 (4 samples with non-SCI and 4 samples with SCI), and GSE52763 (3 samples with non-SCI and 4 samples with SCI). We combined all the data in different samples and used the removeBatchEffect function in the limma 3.62.2 R package (https://www.r-project.org/) to remove batch effects between different sample data. Then limma 3.62.2 R package was employed to identify differentially expressed genes (DEGs) in SCI transcriptome with a false discovery rate (FDR) < 0.05 and a log_2_ fold change (FC) value > 0.585 as filtering conditions. DEGs were visualized using the ggplot2 4.0.0 R package. All databases were accessed on 1 May 2025.

### 2.8. Screening the Target Genes of Pte

The target genes related to Pte were searched for with the keyword “pterostilbene” in a Swiss target prediction database (https://www.swisstargetprediction.ch/), the Traditional Chinese Medicine Systems Pharmacology (TCMSP) database (https://www.tcmsp-e.com/) and PharmMapper (https://www.lilab-ecust.cn/pharmmapper/index.html/). These were merged, and duplications were removed to obtain characteristic genes for Pte. Venny2.1.0 (http://www.liuxiaoyuyuan.cn/) was used for visualization. All databases were accessed on 1 May 2025.

### 2.9. Screening the Characteristic Genes of Oligodendrocytes

Genes related to oligodendrocytes were searched for with the keyword “oligodendrocytes” in GeneCards (https://www.genecards.org/), TTD (https://db.idrblab.net/ttd/), the Online Mendelian Inheritance in Man (OMIM) database (https://www.ncbi.nlm.nih.gov/omim/), Drugbank (https://go.drugbank.com/), and Pharmgkb (https://www.pharmgkb.org/). These were merged, and duplicates were deleted to identify characteristic genes for oligodendrocyte cells. Venny2.1.0 was used for visualization. All databases were accessed on 1 May 2025.

### 2.10. Construction of the Protein–Protein Interaction (PPI) Network

The SCI differential genes, Pte target genes and oligodendrocyte characteristic genes were intercrossed to obtain the common genes of the three. Venny2.1.0 and the pheatmap R package were used for visualization. At the same time, these genes were imported into the STRING platform (https://cn.string-db.org/) to construct a PPI network. The network was visualized using the Cytoscape 3.10.3 software (https://cytoscape.org/), with targets rearranged according to their degree, indicated by gradient color and size variations. All databases were accessed on 1 May 2025.

### 2.11. Screening the Characteristic Genes of PPI Network by Using cytoHubb

With the use of the MCC score, the DMNC score, the MNC score, the Degree score, the Closeness score, the Radiality score, the EPC score, the Betweenness score, and the Stress score in cytoHubb (https://apps.cytoscape.org/download/stats/cytohubba/, accessed on 1 May 2025), the intersection of the top 20 genes was selected as the core genes of the network. Pheatmap 1.0.13 R package was used for visualization.

### 2.12. Cell Culture and Drug Treatment

OLN-93 cells (obtained from ATCC), a permanent oligodendroglia cell line, were cultured in Dulbecco’s modified Eagle’s medium (Hyclone, Logan, UT, USA) containing 10% FBS (Gibco, Grand Island, NY, USA) under 37 °C and 5% CO_2_, and the medium was changed every day. The experiment was divided into four groups: (1) the Control group, for which the cells were not treated with any medications; (2) the Erastin group, for which cells were treated with erastin (10 μM) (HY-15763, MCE, Monmouth Junction, NJ, USA) for 12 h to induce ferroptosis; (3) the Pte-treated group, for which cells were pre-treated with Pte (10 μM) for 12 h, followed by 12 h exposure to erastin (10 μM); and (4) the ML385 group, for which cells were co-treated with Pte (10 μM) and ML385 (5 μM) (HY-100523, MCE, USA) for 12 h to inhibit Nrf2. After that, a change to erastin for 12 h was implemented.

### 2.13. Cell Viability Assay

First, the cell viability of the OLN-93 cells in the Pte (0, 1, 10, 20, and 40 μM) and erastin (0, 0.5, 1, 5, 10, and 20 μM) groups was evaluated using the Cell Counting Kit-8 (CCK-8) (C0038, Beyotime, Shanghai, China) to measure the cytotoxicity of each drug. Briefly, the cells were plated into 96-well plates at 4 × 10^3^ cells per well, with each experimental group containing a minimum of 3 replicate wells, and the cells were incubated for 12 h. When the cell confluence reached about 40%, the medium was replaced with a complete medium containing different concentrations of Pte (0, 1, 10, 20, and 40 μM) or erastin (0, 0.5, 1, 5, 10, and 20 μM), and incubation was continued for 12 h. The CCK-8 working solution was added to all wells, incubating for 30 min at 37 °C, and the OD value was determined at 450 nm. Next, the optimal concentration of Pte for treatment was determined through CCK-8. Specifically, cells were pretreated with Pte (0, 1, 5, and 10 μM) for 12 h, followed by injury with 10 μM erastin for 12 h, after which cell viability was measured. Finally, cells were co-treated with ML385 and Pte prior to erastin treatment, and cell viability was assessed following erastin exposure.

### 2.14. Immunofluorescence

OLN-93 cells were fixed in 4% paraformaldehyde at room temperature for 10 min. After blocking with 10% normal goat serum in PBS containing 0.2% Triton X-100 for 1 h at room temperature, cells or spinal cord sections were incubated with anti-Olig1 (GTX104823, Gene Tex, Irvine, CA, USA, 1:500), anti-GPX4 (GB120010-50, Servicebio, Wuhan, China, 1:500), anti-SLC7A11 (382036, Zenbio, Durham, NC, USA, 1:400), anti-Nrf2 (AF0639, Affinity, San Francisco, CA, USA, 1:500), or anti-Keap1 (MB4221, Bioword, Irving, TX, USA, 1:100) at 4 °C overnight. After three washes of 10 min in PBS, cells or sections were incubated with Alexa Fluor 488 or Alexa Fluor 594-conjugated goat secondary antibody (Proteintech, 1:500) and DAPI (Beyotime, Shanghai, China) for 1 h at room temperature. A Zeiss inverted fluorescence microscope (Axio Imager 2, Carl Zeiss, Oberkochen, Germany) was used to capture representative images.

### 2.15. Western Blot

Total proteins of spinal cord tissue or OLN-93 cells were extracted in RIPA lysis buffer with PSMF (0.1 mM). Nuclear or cytoplasmic protein was extracted from OLN-93 cells using the kit (P0027, Beyotime, Shanghai, China) according to the instructions. The protein concentration was calculated using the BCA protein assay kit (P0010, Beyotime, Shanghai, China). An equal amount of protein (20–40 μg) was electrophoresed in 10% SDS-PAGE gel and then transferred to the PVDF membrane (ISEQ00010, Millipore, Darmstadt, Germany). After the membranes were blocked with 5% non-fat milk in Tris-buffered saline Tween-20 (TBS-T) for 2 h at room temperature, primary antibodies against GPX4 (DF6701, Affinity, 1:2000), SLC7A11 (382036, Zenbio, 1:1000), Nrf2 (16396-1-AP, Proteintech, Rosemont, IL, USA, 1:1000; AF0639, Affinity, 1:2000), Keap1 (BS6783, Bioword, 1:2000), MBP(AF4085, Affinity, 1:1000), and Lamin B1 (BS80098, Bioword, 1:1000) were added and incubated overnight at 4 °C. Suitable secondary antibodies conjugated with horseradish peroxidase (HRP) were employed, and detection was carried out using a chemiluminescence-enhanced chemiluminescence (ECL) kit (P0018S, Beyotime, Shanghai, China).

### 2.16. FerroOrange Detection

OLN-93 cells were seeded in slides with 12-well plates at 1 × 10^4^ cells per well and pretreated with Pte (10 μM) or ML385 (5 μM) for 12 h prior to exposure to erastin (10 μM). After 12 h incubation, 1 μm FerroOrange (F374, Dojindo, Kumamoto, Japan) was added to the culture medium and incubated for 30 min. Representative images were captured with the use of a fluorescence microscope.

### 2.17. Lipid Peroxides Assay

Lipid peroxide levels were measured using the BODIPY™ 581/591 C11 kit (C10445; Waltham, MA, USA). After each group of OLN-93 cells was treated with the drug, the medium was discarded, and the medicated cells were incubated with the BODIPY™ 581/591 C11 probe at a 5 µM concentration. This incubation step was carried out for 30 min in strict accordance with the protocol provided by the kit manufacturer. Following incubation, the fluorescence was detected by fluorescence microscopy.

### 2.18. Transmission Electron Microscopy

After drug treatment, the cell pellets were collected and fixed in 2.5% glutaraldehyde for a duration of 2 h. Then, the cells were fixed with 1% osmium tetroxide at room temperature, with this fixation step lasting 1 h. The fixed cells were subjected to dehydration using a gradient concentration series of acetone and subsequently embedded in epoxy resin. Ultrathin sections were stained with uranium acetate staining solution and lead citrate staining solution. Finally, the ultrastructural features of the cells were observed under a TEM (TECNAI G2 20 TWIN, FEI, Hillsboro, OR, USA).

### 2.19. Molecular Docking (MD)

Nuclear Magnetic Resonance (NMR) structure files of Nrf2 (2LZ1) and X-ray crystallography of Keap1 (2FLU) were retrieved from the Protein Data Bank (PDB) database (https://www.rcsb.org/). The 3D structure of Pte was obtained from the PubChem database (https://pubchem.ncbi.nlm.nih.gov/). Pretreatment procedures and MD operations for Pte and the protein receptors (Nrf2 and Keap1) were carried out using AutoDockTools-1.5.6 software. During this process, the docking binding energy was calculated, and the quality of the docking results was assessed systematically. Subsequently, we used Pymol-2.6 and LigPlus-2.2 software for visualization. All databases were accessed on 1 May 2025.

### 2.20. Molecular Dynamics Simulation (MDS)

The optimal docking model derived from molecular docking (MD) was employed to conduct MDS via the Gromacs-2024.4 software package. To parameterize the receptor proteins, the CHARMM 36 force field and the Transferable Intermolecular Potential with 3 Points (TIP3P) water model were chosen. A cubic box was then constructed to accommodate the receptor protein, after which counterions (Na^+^ or Cl^−^) were added to neutralize the surface charge of the receptor. To achieve the relaxation of all atoms in the receptor protein, energy minimization was performed using the steepest descent and conjugate gradient methods, each set to 10,000 steps. The minimized system was subsequently subjected to gradual heating over 2 ns under a constant volume and temperature (NVT) ensemble at 300 K, followed by a 2 ns simulation under a constant pressure and temperature (NPT) ensemble at 300 K. Ultimately, the molecular dynamics system was run for 200 ns to examine the dynamic binding mode of the complex, with the simulation results visualized using qtgrace-2.6 and Origin-2024 software.

### 2.21. Statistical Analysis

Data analysis was performed using GraphPad Prism 9.5 software. Each experiment was conducted with a minimum of three independent repetitions to ensure reliability. For the assessment of statistical significance, the GraphPad Prism 9.5 was utilized to carry out either two-tailed unpaired Student’s *t*-tests or a one-way analysis of variance (ANOVA), depending on the experimental design. *p* values of <0.05 were defined as representing a statistically significant difference.

## 3. Results

### 3.1. Pte Promotes the Recovery of Hindlimb Motor Capacity in Rats

**Figure 1:**
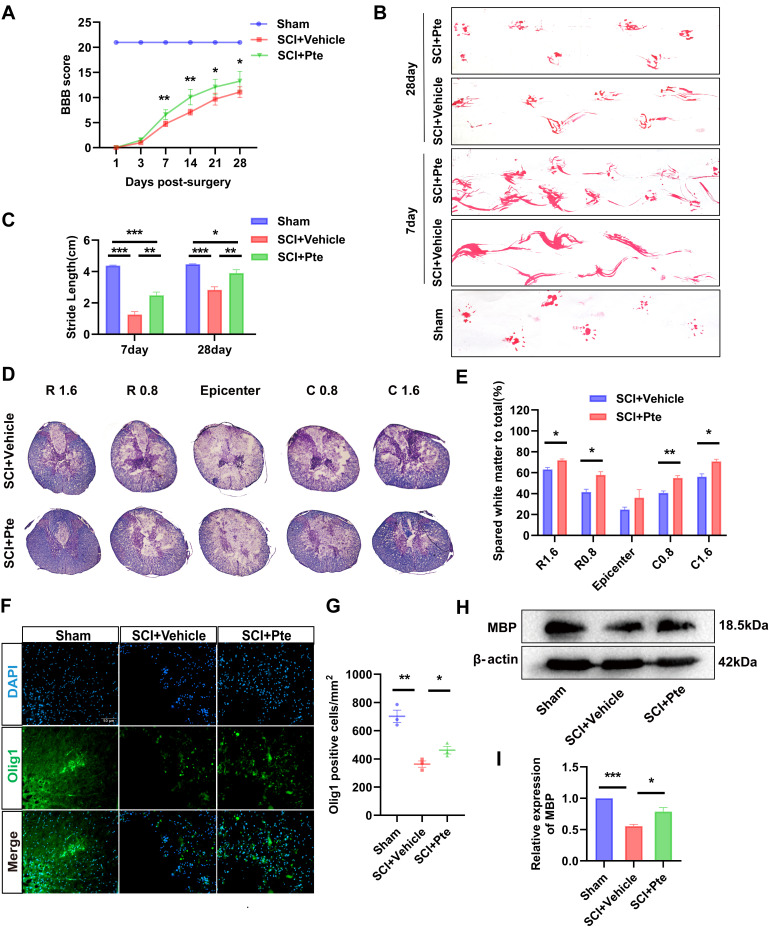
Pte attenuated spinal cord tissue damage and oligodendrocytes loss. (**A**) The BBB scale was employed to assess the recovery of hindlimb motor function over 4 weeks following SCI (*n* = 6). (**B**) Footprint test at 7 and 28 days after SCI (*n* = 6). (**C**) Quantitative analysis of hindlimb adjacent footprints in the footprint test (*n* = 6). (**D**,**E**) The percentages of spared WM areas at 0.8 mm and 1.6 mm rostral and caudal from the epicenter at day 7 after SCI (*n* = 3). (**F**,**G**) IF detected the content of Olig1 and the number of Olig1-positive cells in spinal cord tissues at day 7 after SCI (*n* = 3, scale = 50 μm). (**H**,**I**) The WB results and quantitative analysis of MBP protein expression levels at day 7 after SCI (*n* = 3). Data are expressed as the mean ± SEM. * *p* < 0.05, ** *p* < 0.01, *** *p* < 0.001.

To assess the impact of Pte on locomotor recovery following SCI, we evaluated motor function via the BBB scale and footprint test for rats at designated time points. All animals showed immediate hindlimb paralysis following SCI. BBB was scored 0–21 on day 1 post-injury and then gradually recovered in hindlimb motor function (Figure 1A). At 7–28 days post-injury, the BBB scores of the SCI + Pte group were higher than those of the SCI + Vehicle group (all *p* < 0.05, Figure 1A). At 28 days, the SCI + Vehicle group scored 11.08 ± 1.07, while the SCI + Pte group scored 13.25 ± 1.94. The footprint test was performed on days 7 and 28 post-injury. The result on day 7 showed that the hindlimb function of rats in the SCI + Pte group was significantly restored. In contrast, the rats in the SCI + Vehicle group still failed to raise their hindlimbs. On the 28th day, the hindlimb motor function of rats in both groups was improved, but compared with the SCI + Pte group, the rats in the SCI + Vehicle group still exhibited toe-dragging (Figure 1B). Subsequently, by measuring the length of adjacent fore-and-aft stride lengths in the representative images, it was found that, compared to the Sham group, the stride length of rats was significantly decreased after SCI, but Pte significantly increased the stride length of the rats compared with the SCI + Vehicle group (all *p* < 0.05, Figure 1C). These results indicate that delivery of Pte promotes locomotion recovery in rats after SCI.

### 3.2. Pte Attenuates Spinal Cord Tissue Damage and Oligodendrocyte Loss

To assess the effects of Pte on histopathology after SCI, the myelinated WM was measured by EC staining at day 7 post-injury. The percentages of spared WM areas to the total spinal cord areas were not significantly different between SCI + Pte and SCI + Vehicle rats’ spinal cords at the epicenter (Figure 1D,E). However, the percentages of spared WM were notably increased in the injured spinal cord at 0.8 mm and 1.6 mm rostral and caudal from the epicenter in SCI + Pte rats (all *p* < 0.05, Figure 1D,E). These results show that Pte reduces the loss of residual myelin after SCI. Concurrently, the IF results, with the fluorescence intensity, also showed that the expression of Olig1, the marker of oligodendrocytes, was increased in Pte-treated rats after SCI (all *p* < 0.05, Figure 1F,G). Then, we determined the expression of myelin basic protein (MBP), which serves as a key indicator of mature oligodendrocytes, in spinal cord tissues from SCI rats by WB. Consistent with the IF experiment, the SCI + Pte rats exhibited a significantly increased expression of MBP compared to the SCI + Vehicle rats (all *p* < 0.05, Figure 1H,I). These results suggest that Pte treatment reduces the death of oligodendrocytes, further affirming the neuroprotective effect of Pte after SCI.

### 3.3. Pte Protects Spinal Cord Tissue by Suppressing Ferroptosis

**Figure 2:**
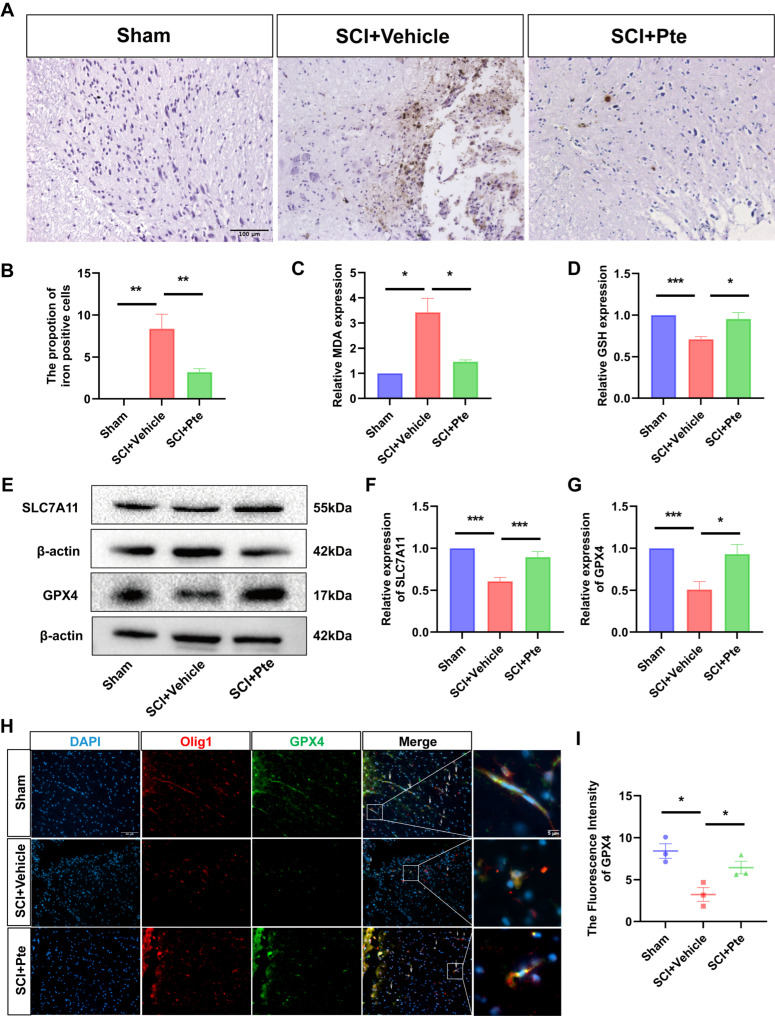
Pte protects spinal cord tissue by suppressing ferroptosis. (**A**) The expression level of iron ions in tissues after 7 days of SCI in different groups (*n* = 3, scale = 100 μm). (**B**) Quantification of iron-positive area (*n* = 3). (**C**,**D**) MDA content and GSH content in tissues 7 days after SCI in different groups (*n* = 3). (**E**–**G**) Protein expression levels of SLC7A11 and GPX4 in tissues 7 days after SCI in different groups (*n* = 3). (**H**,**I**) IF to detect the expression of GPX4 and quantitative analysis in oligodendrocytes in spinal cord tissues (*n* = 3). The white arrows indicated the cells that were double positive for Olig1 and GPX4. Data are expressed as the mean ± SEM. * *p* < 0.05, ** *p* < 0.01, *** *p* < 0.001.

We tested ferroptosis-related molecules in the spinal cords of rats on day 7 post-SCI. Iron deposition is an early signal initiating ferroptosis. In this experiment, Perls blue + DAB staining was used to assess the amount of iron ions in different groups of spinal cord tissues after SCI. As shown in Figure 2A,B, the Sham group exhibited no detectable iron ions, whereas a substantial increase was observed after injury. The number of iron cells in the injured spinal cord was significantly reduced in the SCI + Pte rats compared to those in the SCI + Vehicle rats (all *p* < 0.05, Figure 2A,B).

The production of lipid peroxides is a hallmark of ferroptosis. We further examined the effects of Pte on MDA levels after SCI. The result shows that the MDA levels were markedly higher in the SCI + Vehicle group compared to the Sham group. However, SCI + Pte treatment considerably reduced MDA levels compared to the SCI + Vehicle group (all *p* < 0.05, Figure 2C). Subsequently, we detected the factors related to anti-lipid peroxidation, GSH, SLC7A11 and GPX4. The GSH levels were suppressed in the SCI + Vehicle rats and promoted in the SCI + Pte rats (all *p* < 0.05, Figure 2D). The SLC7A11 and GPX4 were detected by WB in the spinal cords of different groups. The results showed that the expression of GPX4 and SLC7A11 was significantly decreased after SCI. Pte treatment markedly increased the expression of GPX4 and SLC7A11 at the protein level (Figure 2E–G). At the same time, spinal cord tissues were assayed for GPX4 expression in oligodendrocytes by IF double staining, and Olig1 was used to stain oligodendrocytes. The results showed low levels of GPX4 in Olig1 oligodendrocytes of SCI + Vehicle group rats compared to the Sham group, and Pte treatment upregulated the numbers of these cells (Figure 2H,I). These findings suggest that Pte exerts a protective effect on oligodendrocytes by inhibiting ferroptosis after SCI.

### 3.4. Nrf2 Is an Effective Target of Pte

**Figure 3:**
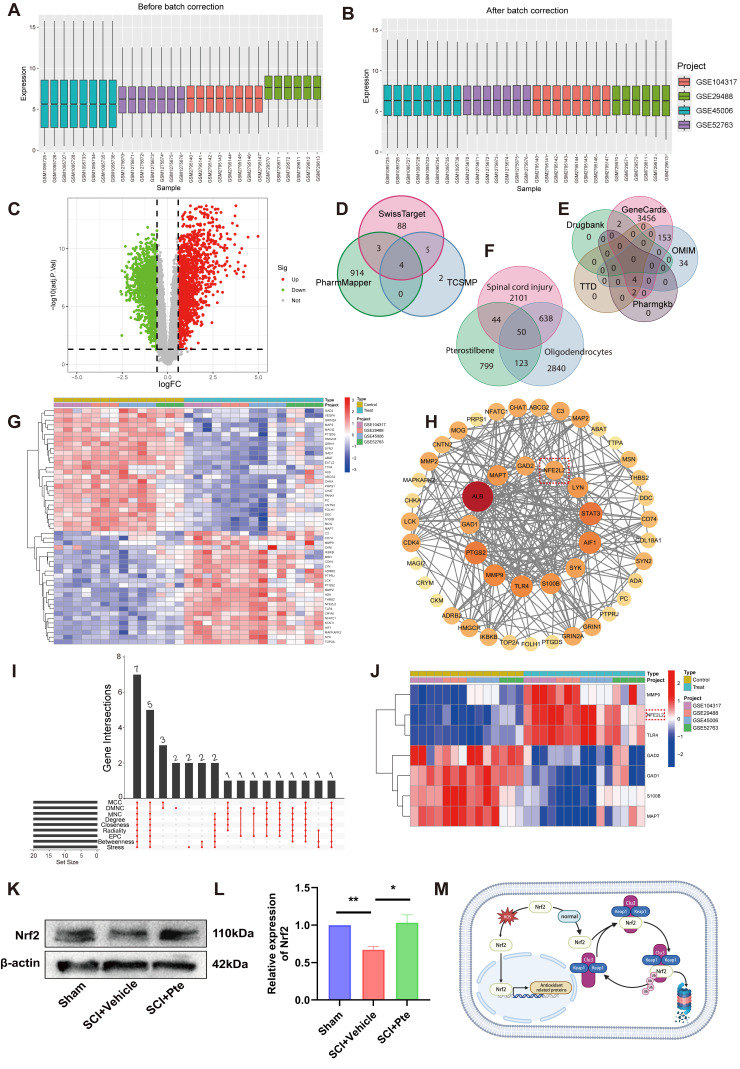
Nrf2 is an effective target of Pte. (**A**,**B**) Bar graphs of gene expression in each group before and after debatching. (**C**) Volcano plot of differentially expressed genes in SCI. (**D**,**E**) Venn map of Pte and oligodendrocytes targets. (**F**) Venn map of SCI, Pte, and oligodendrocytes common targets. (**G**) Heat map of SCI, Pte, and oligodendrocytes intersection genes. (**H**) PPI Network of the 50 common targets. The area within the red dashed box represents the target gene “Nrf2 (*NFE2L2*)”. (**I**) cytoHubb scoring results. (**J**) Heat map of the core genes of the seven networks. (**K**,**L**) Protein expression levels of Nrf2 in tissues 7 days after SCI in each group (* *p* < 0.05, ** *p* < 0.01, *n* = 3). Data are expressed as the mean ± SEM. (**M**) Schematic representation of Nrf2 under normal and oxidative stress conditions.

Nrf2 is a key transcription factor regulating SLC7A11 and GPX4. To predict whether Pte can exert its anti-ferroptotic effects by activating Nrf2, four sets of transcription data of spinal cord tissues from normal and injured rats were obtained from the GEO database (GSE104317, GSE29488, GSE45006, GSE52763). We merged and debatched them (Figure 3A,B), and used FDR < 0.05 and |log2FC| > 0.585 as screening conditions, and we finally obtained 2833 differentially expressed genes (Figure 3C), respectively, from the Swiss Target, TCSMP, PharmMapper, GeneCards, OMIM, Pharmgkb, TDD, and Grugbank database screening of Pte targets (Figure 3D) and genetic characteristics of oligodendrocytes (Figure 3E). Finally, 1016 targets and 3651 characteristic genes were obtained. By intercrossing the SCI differential genes, Pte targets, and oligodendrocyte characteristic genes (Figure 3F), we obtained 50 common targets (Figure 3F,G). These 50 common targets were uploaded to the STRING database to construct a PPI network. Cytoscape 3.10.3 was used to analyze the topology of the PPI network for the intersecting targets. Following the removal of proteins that lacked protein–protein interactions, 48 core targets were identified (Figure 3H). In this visualization, the larger the node, the darker its color and the higher its degree value within the network, all of which signify greater importance. We further used cytoHubb to find the core genes of the network by intersecting the top 20 genes with the highest scores of MCC, DMNC, MNC, Degree, Closeness, EPC, Betweenness, and Stress (Figure 3I). We obtained the core genes of seven networks. We found that Nrf2 (*NFE2L2*) is included among them (Figure 3J). It is highly likely that Nrf2 participates in the anti-ferroptotic process of Pterostilbene. We then performed a WB assay to investigate the effects of Pte on Nrf2 expression in the injured spinal cord of rats. The WB results show that, compared with vehicle treatment rats, Pte upregulated the expression of Nrf2 in the Pte treatment rats (Figure 3K,L, *p* < 0.05). Previous studies have shown that Nrf2 is normally maintained at low levels by ubiquitination degradation when bound to Keap1. However, when cells undergo oxidative stress, Nrf2 will dissociate from Keap1 and further translocate to the nucleus to promote the expression of antioxidant proteins (Figure 3M). Therefore, we hypothesized that Pte promotes Nrf2 nuclear translocation by competitively binding to Keap1 to promote the dissociation of Nrf2 from Keap1 and inhibit the degradation of Nrf2.

### 3.5. Pte Inhibits Erastin-Induced Oligodendroglial Cell Ferroptosis

**Figure 4:**
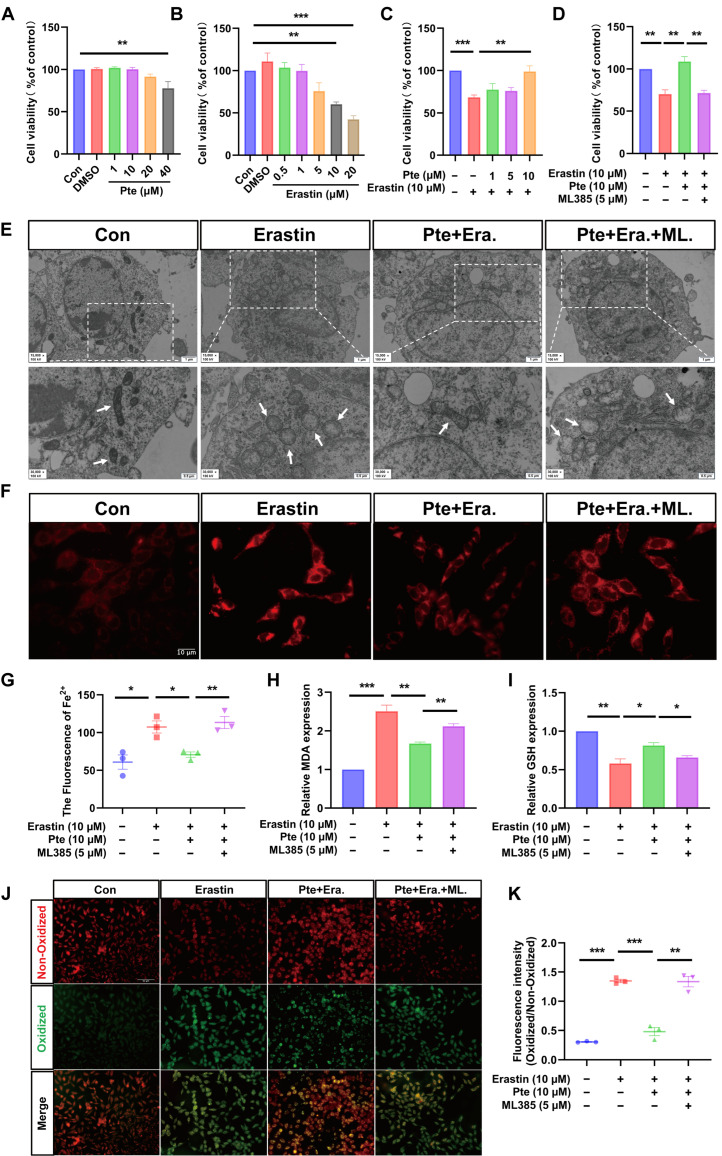
Pte inhibits Erastin-induced oligodendroglial cell ferroptosis. (**A**) CCK-8 for the viability of OLN-93 cells, cultured in medium containing 0, 1, 10, 20, and 40 μM Pte for 12 h (*n* = 3). (**B**) Cell viability of OLN-93 cells was detected after being treated with different concentrations of erastin (0, 0.5, 1, 5, 10, and 20 μM) for 12 h (*n* = 3). (**C**) CCK-8 for the viability of OLN-93 cells, pretreated with different concentrations of Pte (0, 1, 5, and 10 μM) for 12 h, followed by treatment with erastin (10 μM) for 12 h (*n* = 5). (**D**) Cell viability of OLN-93 cells pretreated with Pte (10 μM) and ML385 (5 μM) for 12 h, followed by treatment with erastin (10 μM) for 12 h, was detected (*n* = 3). (**E**) Electron microscopy was used to observe the ultrastructure of mitochondria in each group of cells. (The white arrow indicates the mitochondria of each group, *n* = 3, scale = 1 or 0.5 μm.) (**F**) A FerroOrange fluorescence probe was used to detect the intracellular ferrous ions in each group (*n* = 3, scale = 10 μM). (**G**) FerroOrange fluorescence intensity was quantified (*n* = 3). (**H**,**I**) Assay kits were used to detect the levels of MDA and GSH in each group (*n* = 3). (**J**) The C11 BODIPY 581/591 fluorescent probe was used to detect the degree of lipid peroxides in each group (*n* = 3, scale = 50 μm). (**K**) The C11 BODIPY 581/591 fluorescence intensity was quantitatively analyzed (Oxidized/Non-Oxidized) (*n* = 3). Data are expressed as the mean ± SEM. * *p* < 0.05, ** *p* < 0.01, *** *p* < 0.001.

To investigate whether Pte directly regulates the levels of ferroptosis-associated factors in oligodendrocytes, we treated OLN-93 cells with the ferroptosis inducer erastin to validate the data obtained from in vivo experiments. The results of CCK-8 showed that Pte at concentrations of 1–20 μM did not significantly decrease the viability of OLN-93 cells (all *p* > 0.05, Figure 4A). Meanwhile, we treated OLN-93 cells with different concentrations of erastin for 12 h. The outcomes of the CCK-8 assay revealed that, at an erastin concentration of 10 μM, cell viability decreased to approximately 50% (*p* < 0.01, Figure 4B). Thus, OLN-93 cells were pretreated with 1, 5 and 10 μM of Pte for 12 h, followed by treatment with 10 μM erastin for another 12 h. Cell viability was then assayed to observe the effect of Pte on inhibiting ferroptosis. The results demonstrated that 10 μM Pte effectively suppressed erastin-induced cell death (*p* < 0.01, Figure 4C). Thus, we chose 10 μM of Pte for subsequent experiments.

Based on in vivo experimental findings demonstrating that Pte can activate the expression of Nrf2, we treated OLN-93 cells with the Nrf2-specific inhibitor ML385 to confirm whether Pte suppresses ferroptosis through the activation of Nrf2. The CCK-8 results show that the addition of ML385 counteracts the effects caused by Pte (*p* < 0.01, Figure 4D).

TEM observation results showed that the mitochondrial structures in the control group cells were intact. Compared with the control group, most of the mitochondrial cristae in the Erastin group were reduced or disappeared, and the outer membranes were ruptured. In comparison to the Erastin group, the above mitochondrial abnormalities were all improved in the Pte + Era. group. However, in the Pte + Era. + ML. group, there was an increase in the reduction of cristae and disruption of the outer membrane in mitochondria (Figure 4E). Using the FerroOrange probe to measure Fe^2+^ content in OLN-93 cells, the results showed that the IF intensity in the Pte + Era. group was significantly lower than that in the Erastin group, while the addition of ML385 increased the fluorescence level (all *p* < 0.05, Figure 4F,G). Then, we found that Pte treatment significantly suppressed MDA levels (*p* < 0.01) (Figure 4H) and increased GSH levels (*p* < 0.05) (Figure 4I) in erastin-stimulated OLN-93 cells. BODIPY 581/591 C11 staining demonstrated that Pte reduced erastin-induced lipid ROS production in OLN-93 cells (*p* < 0.001) (Figure 4J,K). Moreover, these effects were reversed by ML385 (*p* < 0.05 or *p* < 0.01, Figure 4G–K), indicating that Pte mitigated Erastin-induced cellular injury by enhancing Nrf2 activation, thereby suppressing ferroptosis.

### 3.6. MD and MDS Show That Pte Had a Higher Affinity for Keap1

**Figure 5:**
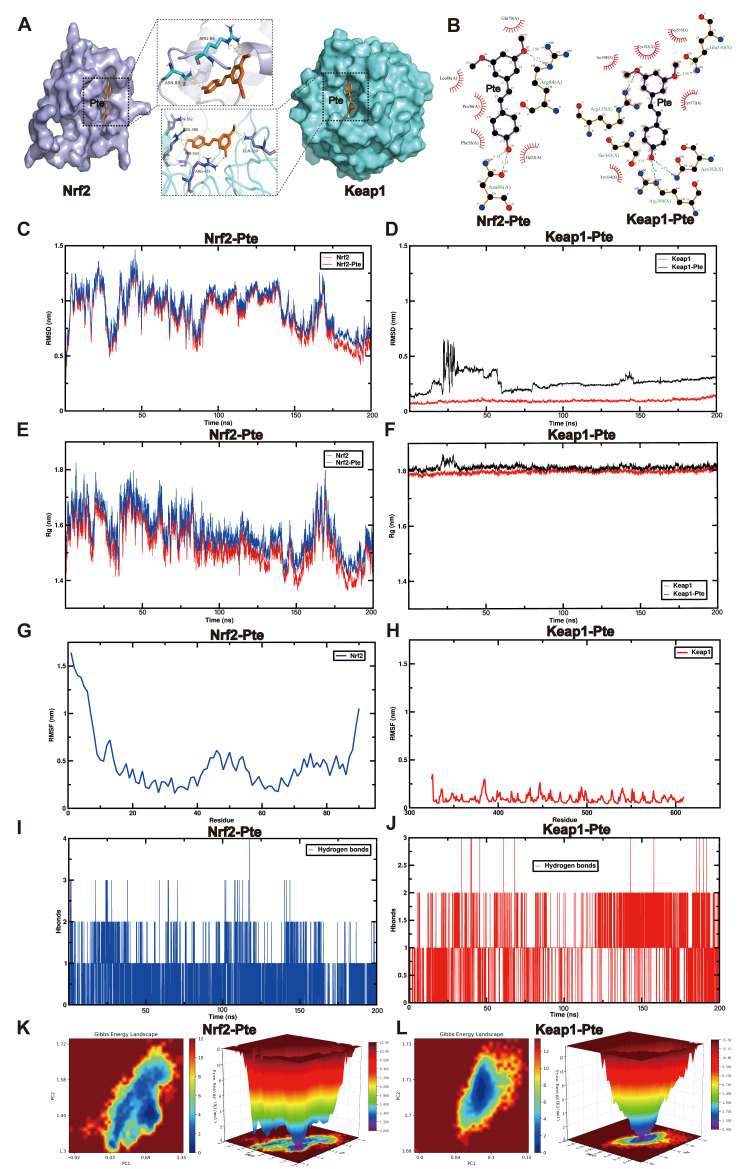
MD and MDS showed that Pte had a higher affinity for Keap1. (**A**) 3D binding mode of Nrf2-Pte and Keap1-Pte. (**B**) 2D binding mode of Nrf2-Pte and Keap1-Pte. (**C**,**D**) RMSD of Nrf2, Keap1, and their complexes formed with Pte, indicating the stability of proteins and complexes across various time points. (**E**,**F**) Rg of Nrf2, Keap1, and their complexes formed with Pte, indicating the fixity of proteins and complexes. (**G**,**H**) RMSF of Nrf2 and Keap1, demonstrating the flexibility of each residue on these two proteins. (**I**,**J**) Hbonds number of complexes Nrf2-Pte and Keap1-Pte, showing hydrogen bonds formed across various time points. (**K**,**L**) 2D and 3D mapping of the free energy landscape of Nrf2-Pte and Keap1-Pte. RMSD: root-mean-square deviation; Rg: radius of gyration; RMSF: root-mean-square fluctuation; PC: principal component.

To further test our hypothesis that Nrf2 activation by Pte does not directly bind to Nrf2 but through competitive binding to Keap1, we employed MD and MDS. MD was performed using AutoDockTools (version 1.5.6) to evaluate the binding energies and hydrogen bonding interactions of Nrf2-Pte and Keap1-Pte. The MD results showed that Nrf2-Pte formed four hydrogen bonds with a binding energy of −5.4 kcal/mol. Keap1-Pte formed five hydrogen bonds with a binding energy of −6.8 kcal/mol (Figure 5A,B). Apparently, Pte forms more hydrogen bonds with Keap1 and has a lower binding energy than Nrf2. It is noteworthy that Pte docking with Keap1 happens to occupy the active pocket where Keap1 normally binds to Nrf2 (Figure S1A–C).

We used MDS for further exploration. Root-mean-square deviation (RMSD) can be used as a powerful measure to evaluate the stability and position deviation from the initial conformation of a protein and ligand after binding. The RMSD with less fluctuation indicates a strong conformational stability of the system after it reaches equilibrium. Our results showed that the RMSD values of Nrf2-Pte and Keap1-Pte were within the normal range (Figure 5C,D), while Keap1-Pte reached equilibrium in a shorter time and exhibited stronger conformational stability. The radius of gyration (Rg) (Figure 5E,F; Figure S1D–G) and the solvent-accessible surface area (SASA) (Figure S1H,I) also indicate that Keap1-Pte fluctuates less during the simulation and maintains better compactability and stability.

Root-mean-square fluctuation (RMSF) shows the flexible movement and structural changes of specific residues in the protein during the simulation. We found that Nrf2-Pte exhibited significantly higher flexibility than Keap1-Pte (Figure 5G,H), indicating that Nrf2 maintained a less stable secondary structure during the simulation, whereas Keap1 maintained a more stable conformation. Hydrogen bonds directly reflect the degree of stability of protein and ligand binding. The Nrf2-Pte complex shows 0–4 hydrogen bonds (mainly 1) (Figure 5I), whereas the Keap1-Pte complex shows 0–3 hydrogen bonds (mainly 2) (Figure 5J). Interestingly, the molecular dynamics results and MD junctions were not consistent in terms of the number of hydrogen bonds, which may be related to the different definition of hydrogen bonds for different analytical tools, but in general, Keap1-Pte formed more hydrogen bonds and showed stronger stability.

Principal component analysis (PCA) was used to extract the first two principal components from RMSD and Rg. Together with the Gibbs relative free energies, these main components form the 2D and 3D free energy landscapes (Figure 5K,L). The 3D free energy landscape of Keap1-Pte shows a unique and distinct minimum energy cluster, indicating that the complex fluctuates around a single stable conformation throughout the simulation without significant conformational transitions or dissociation, whereas Nrf2-Pte shows subminima, possibly corresponding to partially dissociable or weakly bound conformations. In addition, Keap1-Pte exhibited a larger depth and smaller width than Nrf2-Pte, further indicating that Keap1-Pte had a more stable conformation.

### 3.7. Pte Increases Nrf2 Nuclear Translocation by Promoting the Dissociation of Keap1 and Nrf2, Thereby Activating the Nrf2/SLC7A11/GPX4 Axis

**Figure 6:**
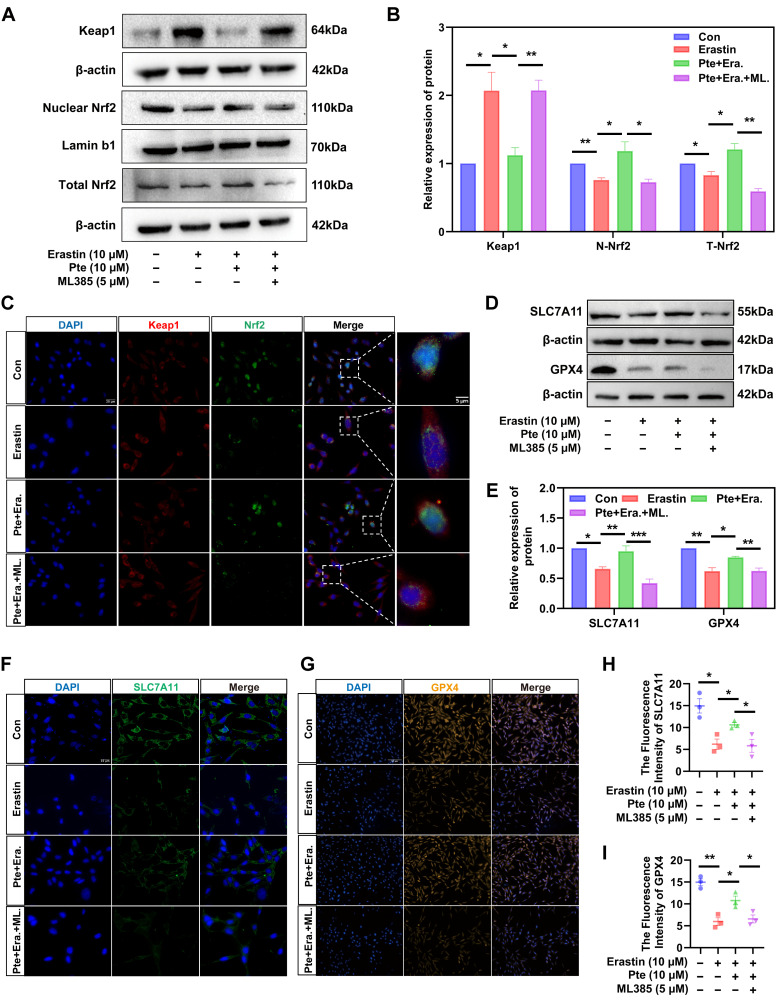
Pte increases Nrf2 nuclear translocation by promoting the dissociation of Keap1 and Nrf2, thereby activating the Nrf2/SLC7A11/GPX4 axis. (**A**,**B**) WB results and quantitative analysis of the expression levels of Nrf2 (nuclear protein and total protein) and Keap1 in each group (*n* = 3). (**C**) IF staining was used to evaluate the nuclear translocation of Nrf2 in each group (*n* = 3). (**D**,**E**) The WB results and quantitative analysis of SLC7A11 and GPX4 protein expression levels in each group of cells (*n* = 3). (**F**–**I**) The IF detection of GPX4 and SLC7A11 expression levels in each group of cells (*n* = 3). Data are expressed as the mean ± SEM. * *p* < 0.05, ** *p* < 0.01, *** *p* < 0.001.

WB and IF staining of Nrf2 showed that the nuclear translocation of Nrf2 was reduced in the Erastin group, while it was enhanced in the Pte group, and this effect was inhibited by ML385 (Figure 6A–C). It was also observed that the Keap1 content was increased and the Nrf2 content was decreased through erastin treatment, which was reversed through Pte treatment (Figure 6A,B). This is consistent with our previous hypothesis that Pte inhibits ferroptosis by promoting the dissociation of Nrf2 from Keap1 and its translocation into the nucleus. We further investigated the expression of Nrf2 downstream anti-ferroptosis proteins GPX4 and SLC7A11. Our findings showed that erastin decreased the levels of SLC7A11 and GPX4 in OLN-93 cells, whereas Pte elevated the expression of these proteins (Figure 6D,E). Further IF staining revealed that the levels of SLC7A11 and GPX4 in OLN-93 cells of the Pte-treated group increased compared to the Erastin group, while ML385 reversed this effect (Figure 6F–I). Taken together, these results suggest that Pte inhibits ferroptosis by promoting the dissociation of Keap1 from Nrf2 to increase Nrf2 nuclear translocation, which then activates the Nrf2/SLC7A11/GPX4 axis.

## 4. Discussion

**Figure 7:**
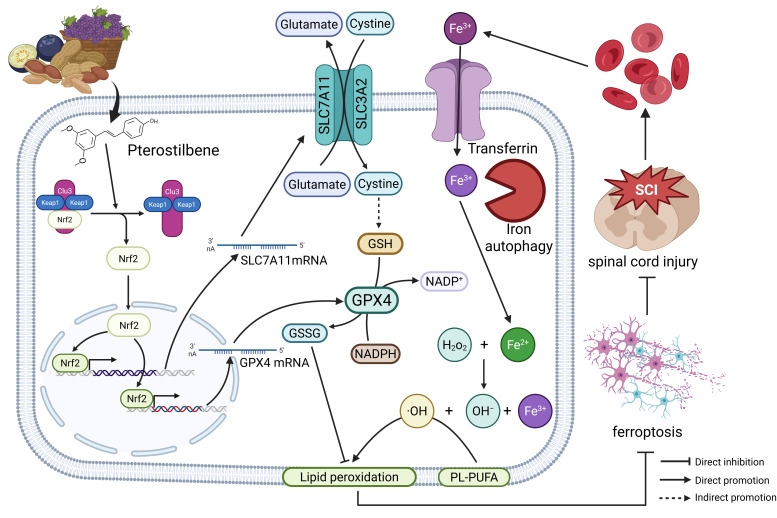
Pte reduces lipid peroxidation by up-regulating the Keap1/Nrf2/SLC7A11/GPX4 axis, which reduces the development of ferroptosis in oligodendrocytes and improves locomotor function in rats with SCI. SLC3A2, Solute carrier family 3 member 2; SLC7A11, Golute Carrier Family 7 Member 11; Nrf2, Nuclear factor E2; Keap1, Kelch Ech-associated protein 1; GSSG, synthesized oxidized glutathione; GSH, Glutshione; NADP^+^, Nicotinamide Adenine Dinucleotide Phosphate; NADPH, Nicotinamide Adenine Dinucleotide Phosphate Hydrogen; Cul3, Cullin-3.

In this study, we demonstrated for the first time that Pte attenuates demyelination due to secondary damage to oligodendrocytes by inhibiting lipid peroxides production and iron deposition after SCI, as well as enhancing the antioxidant system, including GSH, SLC7A11 and GPX4, thereby improving motility and tissue recovery. Additionally, in vitro studies have shown that the Keap1/Nrf2/SLC7A11/GPX4 axis is essential for Pte-mediated anti-oligodendrocyte ferroptosis (Figure 7).

Spinal cord injury (SCI) is a serious disease of the CNS, which usually leads to lifelong disability and severely impairs the quality of life in patients. SCI occurs in two phases: the primary injury caused by physical factors is typically irreversible. Secondary injury, driven by factors such as cell death, edema, glutamate excitotoxicity, inflammation, and lipid peroxidation, exacerbates the severity of SCI. However, secondary SCI can be mitigated or even halted through active therapeutic intervention [1,3]. Therefore, understanding secondary injury represents a significant advancement in the field of SCI treatment. As cells specialized in forming myelin sheaths around neuronal axons, oligodendrocytes play a crucial role in the recovery process following SCI. After SCI occurs, oligodendrocytes are highly susceptible to oxidative stress-induced damage and death. This process triggers secondary injury, accelerating axonal demyelination [8,9]. Therefore, protecting oligodendrocytes and reducing cell death may improve SCI prognosis.

The clinical treatment of SCI mainly includes surgical decompression, hemodynamic support, and drug therapy [36]. The current drug treatment is mainly corticosteroids, which can improve the prognosis of patients with SCI, but they will bring many side effects, such as infection, hyperglycemia, gastrointestinal hemorrhage, and even mortality [37]. Therefore, the development of novel therapeutic agents featuring enhanced efficacy and minimized side effects remains crucial for achieving long-term recovery after SCI. Pterostilbene, a natural dimethyl analogue of resveratrol, has higher bioavailability and pharmacological effects compared to resveratrol due to its two methoxyl groups (-OCH3) [28,29,38]. Pte exhibits multiple biological activities, including anti-inflammatory, anti-glycation, antioxidant, and neuroprotective effects. Several studies have documented Pte’s inhibitory effects on oxidative stress and inflammation. For instance, it demonstrates regulatory functions in conditions such as models of amyotrophic lateral sclerosis, cognitive impairment, inflammatory bowel disease, and atopic dermatitis [33,34,39,40]. It is worth noting that, in terms of drug safety, animal models in preclinical experiments and human experimental data in clinical trials have shown that large doses of Pte do not cause significant toxic effects on the body, which provides an important theoretical basis for the long-term application of Pte in the treatment of SCI [41,42].In this study, we employed a spinal cord injury (SCI) model in rats generated using a spinal cord injury impactor. Following previous research protocols [43,44,45], we administered Pte (40 mg/kg) within 30 min post-injury to investigate its therapeutic efficacy in the rat SCI model. First, we observed BBB scores at different time points in SCI rats. Starting from the 7th day, the SCI + Pte group scored significantly higher than the SCI + Vehicle group. On day 28, the SCI + Pte group scored approximately 13, while the SCI + Vehicle group scored 11, indicating that Pte promotes spinal cord injury recovery. However, the scoring difference diminished at days 21 and 28. The reasons may be as follows. 1. Natural recovery in the model group: As shown in our Figure 1A, the Vehicle group exhibited some natural recovery in the later stages. This is a known phenomenon in SCI rodent models, primarily attributed to compensatory mechanisms and spontaneous plasticity. This naturally narrows the absolute score gap with the treatment groups to some extent. 2. An earlier or higher plateau in the Treatment group: A more significant interpretation is that the SCI + Pte group may have reached a functional recovery plateau earlier, and this plateau level was significantly higher than that of the SCI + Vehicle group. This suggests that Pte did not inhibit the natural recovery process but, rather, significantly enhanced recovery scores and accelerated the recovery process. Therefore, the observations from the initial 28 days sufficiently demonstrate Pte’s motor recovery-promoting function. Similarly, the footprint test at days 7 and 28 also revealed significantly enhanced motor function in the SCI + Pte group. Subsequently, we examined the effects of Pte on the histopathology of SCI rats. The results from EC staining, Olig1 immunofluorescence, and MBP protein detection indicated that Pte treatment reduced loss of central white matter and decreased death of damaged area oligodendrocytes in the spinal cord. These findings suggest Pte possesses neuroprotective effects.

Apoptosis has long been regarded as a dominant mode of cell death in the pathological process of SCI [46]. However, recent studies indicate that, following SCI, hemorrhage in the injured area and surrounding tissues leads to elevated iron ion concentrations at the injury site [47]. This exacerbates oxidative stress and lipid peroxide accumulation, demonstrating that ferroptosis also contributes to the pathophysiological process of SCI, with the most severe effects occurring during the acute phase [48,49]. Therefore, in the current study, we chose to observe the expression of ferroptosis-related factors at day 7 after SCI in rats. This study detected iron ions and lipid peroxide levels in spinal cord tissue using Perls Prussian blue staining and the MDA assay kit. Our experimental results indicate that Pte effectively reduces iron ion accumulation and MDA levels at the site of spinal cord injury, demonstrating anti-ferroptotic effects.

In regulating ferroptosis, the antioxidant factors System Xc-, GSH, and GPX4 play crucial roles. System Xc-, composed of SLC7A11 and SLC3A2, is responsible for transporting cysteine, the precursor for GSH synthesis, into cells [50]. GSH, as a crucial cofactor for GPX4, is essential for maintaining GPX4 activity and expression levels. GPX4 effectively suppresses lipid peroxidation. GPX4 uses GSH as a substrate to synthesize oxidized glutathione (GSSG), which further interferes with the chain reaction of free radicals by converting activated phospholipid hydroperoxide (PLOOH) to inactivated phosphatidylcholine (PLOH) to eliminate PLOOH of polyunsaturated fatty acids (PUFAs). It inhibits the process of lipid peroxidation and, ultimately, ferroptosis. In recent years, it has been recognized as a key regulator of ferroptosis [51,52]. In this study, we found that Pte upregulates the expression levels of SLC7A11, GSH, and GPX4 in the SCI rat model, enhancing the rats’ endogenous antioxidant capacity.

Previous studies have demonstrated that the enzymes GPX4 and SLC7A11, which participate in GSH biosynthesis, are regulated by the transcription factor Nrf2. Nrf2, as a core regulatory element in the oxidative stress defense mechanism, is strictly regulated by the tumor suppressor protein Keap1 ubiquitination pathway and maintained at a low level in the normal state [53,54]. However, in response to oxidative stress, Nrf2 will be liberated from Keap1 and further translocated to the nucleus, triggering the transcriptional activity of a series of genes, including antioxidant response elements (AREs). AREs are a crucial cis-acting element regulating the expression of cellular antioxidant genes, responding to oxidative stress and protecting cells from damage, including SLC7A11 and GPX4, which have a critical function in inhibiting lipid peroxidation and preventing ferroptosis [55,56]. Therefore, we speculate that Pte inhibits the ferroptosis of oligodendrocytes after SCI by promoting the dissociation of Nrf2 from Keap1 and promoting Nrf2 nuclear translocation to activate SLC7A11 and GPX4 transcription. By analyzing the 7-day spinal cord transcriptome data of rats with SCI, we identified the characteristic genes of spinal cord injury. Through the method of network pharmacology, we found that Nrf2 might be the target of Pte. To further validate whether Pte exerts its anti-ferroptotic effects through Nrf2, we established an in vitro ferroptosis model by treating the oligodendrocyte lineage OLN-93 cells with the ferroptosis inducer erastin, followed by treatment with the Nrf2 inhibitor ML385 for in-depth validation. In vitro results demonstrated that Pte mitigates mitochondrial damage, lipid peroxidation, and ferrous ion accumulation in OLN-93 cells. This therapeutic effect was simultaneously suppressed by ML385, indicating that Pte’s neuroprotective action against ferroptosis is associated with Nrf2. At the same time, we hypothesized that Pte might not directly bind to Nrf2. Instead, it might promote the dissociation of Nrf2 from Keap1 and facilitate Nrf2 nuclear translocation to activate the transcription of SLC7A11 and GPX4, thereby inhibiting the ferroptosis of oligodendrocytes after SCI. Our MD results indicated that the binding energy of Pte with Keap1 was lower, and more hydrogen bonds were formed. The MDS results showed that the fluctuation of Pte-Keap1 binding was smaller, the system was more stable, and the binding energy conformation was more stable. It is noteworthy that Pte might promote the dissociation of Keap1 and Nrf2 by occupying the active pocket where Keap1 binds with Nrf2, and increase Nrf2 nuclear import. At the same time, IF and WB experiments demonstrated that Pte promoted the dissociation of Nrf2 from Keap1, facilitated Nrf2 nuclear translocation, and increased the expression of GPX4 and SLC7A11. Therefore, we demonstrated that Pte activates the Nrf2/SLC7A11/GPX4 axis to protect against ferroptosis by promoting the dissociation of Keap1 from Nrf2.

This study involved certain limitations. First, the agent of Pte (40 mg/kg) used in this experiment was determined based on previous literature reports [43,44,45] and preliminary experiments, and there is a lack of pharmacokinetic data for Pte in rats. Secondly, our experiment lacked a positive control drug for treating spinal cord injury (such as methylprednisolone). However, we compared the effect of Pte on improving BBB scores with results from studies using methylprednisolone in similar SCI models [57]. We found that Pte achieved a level of improvement comparable to methylprednisolone (at 28 days, methylprednisolone scored 15 points while Pte scored 13 points; with both control groups scoring 10 points), indirectly indicating that Pte possesses significant and comparable therapeutic potential for SCI treatment. However, incorporating a positive control drug in future studies is essential. In addition, we only observed the recovery of motor function in rats within 28 days, and the recovery of SCI is a long process, so there is a lack of longer-term observations. For ferroptosis, we only studied oligodendrocytes and not other nerve cells. In the future, the therapeutic effect of Pte in different nerve cells can be explored.

## 5. Conclusions

Pte reduces lipid peroxidation via the Keap1/Nrf2/SLC7A11/GPX4 axis, which reduces the development of ferroptosis in oligodendrocytes and improves locomotor function in rats with SCI. The mechanism of action of Pte in inhibiting ferroptosis was elucidated, which provided an experimental foundation for subsequent clinical translational research.

## Data Availability

The original contributions presented in this study are included in the article/Supplementary Material. Further inquiries can be directed to the corresponding author.

## Supplementary Materials

The following supporting information can be downloaded at https://www.mdpi.com/article/10.3390/antiox15020188/s1, Figure S1. (A–C) 3D binding mode of Keap1-Pte and Keap1-Nrf2. (D–G) Rg of Nrf2, Keap1 and their complexes with Pte indicate the fluctuations of proteins and complexes on the x,y, and z axes. (H–I) SASA of Nrf2-Pte and Keap1-Pte, indicating the surface area of each atom or residue in the complex exposed to solvent. Rg: radius of gyration; solve-accessible surface area (SASA).

## Abbreviations

The following abbreviations are used in this manuscript:

SCI	Spinal cord injury
Pte	Pterostilbene
CNS	Central nervous system
GPX4	Glutathione peroxidase
SLC7A11	Golute Carrier Family 7 Member 11
Nrf2	Nuclear factor E2
WM	White matter
EC	Iron-eriochrome cyanine R
BBB	Basso–Beattie–Bresnahan
GEO	Gene expression omnibus
TCMSP	Traditional Chinese Medicine Systems Pharmacology
PPI	Protein-protein interaction
MD	Molecular docking
MDS	Molecular dynamics simulation
RMSD	Root-mean-square deviation
Rg	Radius of gyration
SASA	Solve-accessible surface area
RMSF	Root-mean-square fluctuation
PCA	Principal component analysis
MDA	Malondialdehyde
GSH	Glutathione
FDR	False Discovery Rate
PDB	Protein data bank
TIP3P	Potential with 3 points
NVT	Normal volume and temperature
NPT	Normal pressure and temperature
FC	Log2 fold change
MBP	Myelin basic protein
Keap1	Kelch Ech-associated protein 1
GSSG	Synthesize oxidized glutathione
PLOOH	Phospholipid hydroperoxide
PLOH	Phosphatidylcholine
PUFAs	Polyunsaturated fatty acids
SLC3A2	Solute carrier family 3 member 2
AREs	Antioxidant response elements
