# Supplementary material for: Pterostilbene Promotes Spinal Cord Injury Recovery by Inhibiting Ferroptosis via Keap1/Nrf2/SLC7A11/GPX4 Axis Activation
Source: Antioxidants (Basel). 2026 Feb 2;15(2):188. doi: 10.3390/antiox15020188 (PMC12938647; doi:10.3390/antiox15020188)
Supplement: Supplementary file 1 [file antioxidants-15-00188-s001.zip › antioxidants-4017617-supplementary.pdf]

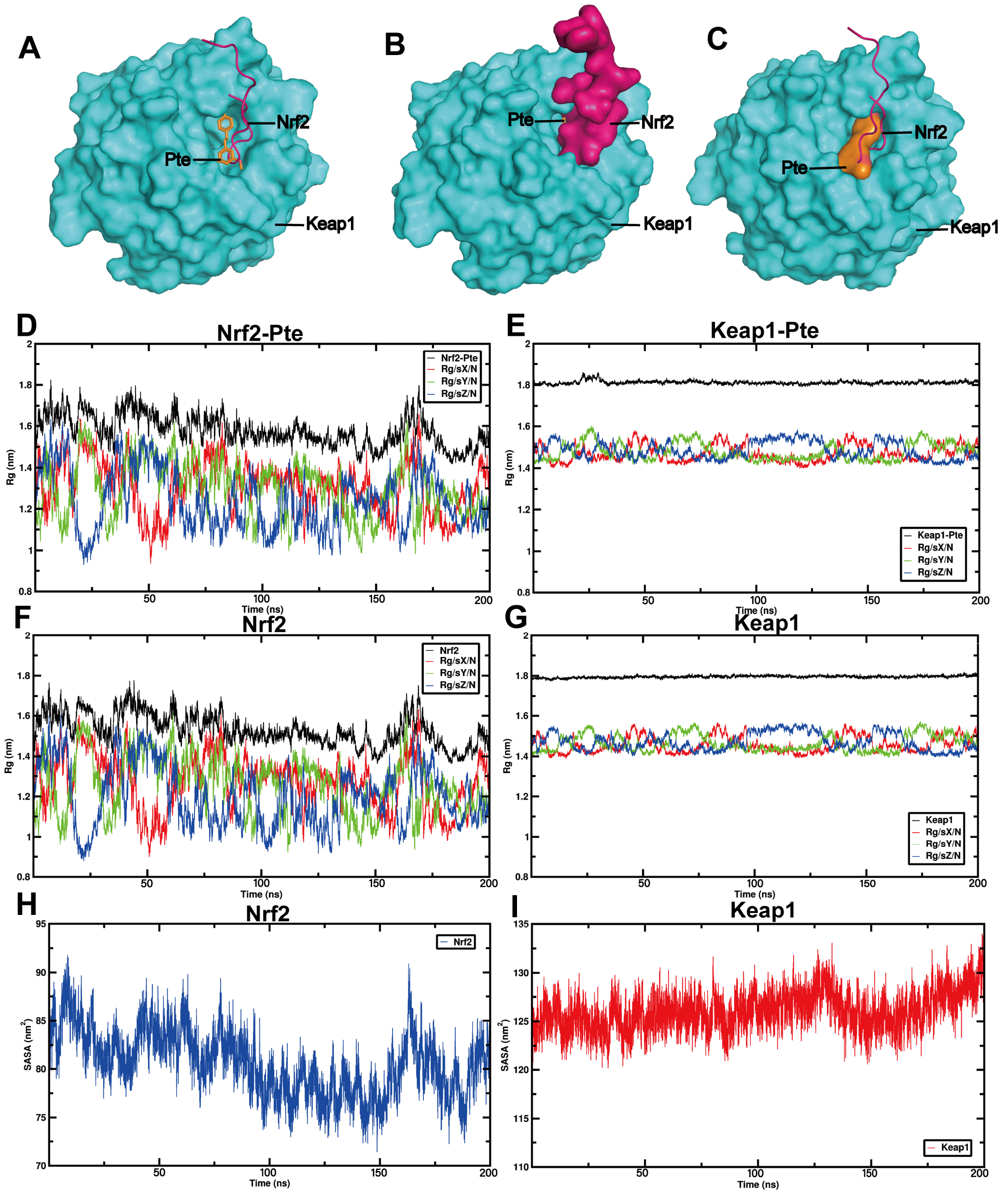

Figure S1. (A-C) 3D binding mode of Keap1-Pte and Keap1-Nrf2. (D-G) Rg of Nrf2, Keap1 and their complexes with Pte indicate the fluctuations of proteins and complexes on the x,y, and z axes. (H-I) SASA of Nrf2-Pte and Keap1-Pte, indicating the surface area of each atom or residue in the complex exposed to solvent. Rg: radius of gyration; solve-accessible surface area (SASA).
